# Effects measurement and spatial differentiation in synergy between pollution and carbon reduction in national urban agglomerations

**DOI:** 10.1371/journal.pone.0289801

**Published:** 2023-09-21

**Authors:** Peijiong Feng, Tianyun Lu, Yaguai Yu, Ruiyan Gao, Taohan Ni

**Affiliations:** 1 School of Economics and Management, Tongji University, Shanghai, China; 2 Business School, Ningbo University, Ningbo, China; 3 Donghai Academy, Ningbo University, Ningbo, China; 4 Business School, The University of Nottingham Ningbo China, Ningbo, China; Shandong University, CHINA

## Abstract

Five national urban agglomerations are selected according to the Fourteenth Five-Year Plan, namely the Yangtze River Delta, the Pearl River Delta, Beijing-Tianjin-Hebei, the Middle reaches of the Yangtze River, and Chengdu-Chongqing. The study on synergistic effects of such national strategic planning urban agglomerations, defined as coordinated degrees, and their time-series trends from 2016 to 2021 is significant for the practice of the national "double carbon" goal. Spatial differentiation of coordinated degree in five agglomerations is analyzed based on the Theil Index, along with regional linkage strength of coordinated degree under the gravity model. Conclusions include: (1) a downward trend is shown from 2016 to 2021 for the coordinated degree, along with the Pearl River Delta is the best among the five agglomerations; (2) the Middle reaches of the Yangtze River is the highest, followed by the Pearl River Delta and the Yangtze River Delta of coordinated degree in 2021; (3) the main cause of downwards in coordinated degree combined by a stable decline in carbon emissions and fluctuating increase in pollutants; (4) intra-regional differences in the Yangtze River Delta and the Middle reaches of the Yangtze River are relatively large, compared with the smallest in the Pearl River Delta measured by Theil Index; (5) coefficients of variation are relatively higher in the Middle reaches of the Yangtze River and the Yangtze River Delta, followed by the Beijing-Tianjin-Hebei, the Pearl River Delta and Sichuan-Chongqing urban. Consequently, countermeasures are proposed from the perspective of government, including technology and regional cooperation, policy innovation by overall coordination, synergy promotion by technological innovation, and regional synergy by win-win cooperation.

## Introduction

The CPC Central Committee and The State Council issued Opinions of The State Council on the Complete, Accurate, and Comprehensive Implementation of the new Development Concepts and the Work of Achieving Peak Carbon Neutrality on September 22, 2021 [1], putting forward the strategic goals of building a green, low-carbon, circular development economic system and a clean, low-carbon, safe and efficient energy system. On June 13, 2022, the Ministry of Ecology and Environment and seven other ministries and commissions jointly issued the Implementation Plan for the Synergy of Pollution and Carbon Reduction [2], requiring that "the realization of the synergy between Pollution and Carbon Reduction is ought to be the overall focus of promoting the comprehensive green transformation of economic and social development". While the situation of air pollution prevention is still serious in China, to achieve the goals of "Carbon Peak, Carbon Neutrality" and "Synergy between Pollution and Carbon Reduction" means that the construction of ecological civilization in China needs to enter a new stage.

Urban agglomerations is the spatial main body of the national promotion of new-type urbanization construction, and it is also a sensitive area where ecological and environmental problems are highly concentrated and intensified [3]. With good stability, sustainability, and the ability to maintain, restore, and upgrading of the functional structure, urban agglomerations break the limit of scale in a larger scope and release an organizational system with huge economic, environmental, and social values beyond the reach of a single city [4]. In the Outline of the Fourteenth Five-Year Plan for National Economic and Social Development of the People’s Republic of China and the Vision 2035 [5], The government proposes to concentrate on the construction of intercity transportation networks in the five national urban agglomerations, namely the Yangtze River Delta, Pearl River Delta, Beijing-Tianjin-Hebei, the Middle reaches of the Yangtze River and Chengdu-Chongqing urban agglomerations. Therefore, they are selected as the research objects. The five national urban agglomerations are relatively mature and are increasingly prominent in the national regional strategy. Currently, the development of the Beijing-Tianjin-Hebei urban agglomeration is mainly oriented to traditional manufacturing and heavy chemical industries, and the core cities do not have an obvious drive on regional development; the Yangtze River Delta and Pearl River Delta urban agglomerations are directed by industrial transformation and upgrading needs, vigorously developing modern service industries and cultivating new industries; the Chengdu-Chongqing urban agglomeration and the Middle reaches of the Yangtze River urban agglomeration focus on developing advanced manufacturing industries and enhancing regional core competitiveness. The five national urban agglomerations’ collaborative governance systems for Pollution and Carbon Reduction are analyzed and future improvement directions are propounded, to facilitate the coordination and interaction of industrial division of infrastructure and environmental governance among trans-regional cities.

## Literature review

The essence of collaborative governance of Pollution and Carbon Reduction is the transformation of ecological environment governance from "pollution first, treatment later" to "prevention at source" and "treatment at source". Guided by high-quality economic development, various measures are adopted to promote the transformation and development of Pollution and Carbon Reduction and accelerate the formation of resource-saving and environment-friendly industrial structures, energy structures, building structures, and transportation spatial patterns. Currently, domestic and foreign scholars are attaching more significance to the collaborative governance of Pollution and Carbon Reduction. In the research field, the research has been limited to the field of carbon emission since the early stage, including the coupling coordination between carbon emission intensity and high-quality economic development of urban agglomeration in the Middle reaches of the Yangtze River [6], the spatial difference and spatial convergence of provincial carbon emission efficiency [7]; Or studies limited to the field of air pollutants, with the effects of six pollutant concentrations (CO, NO2, O3, PM10, PM2.5, SO2) on the spatial evolution characteristics of Chinese cities [8] as a typical representative. Recent studies have further shifted their perspective to the field of Synergy Between Pollution and Carbon Reduction, from defining the concept of "Coordinated Degree of Pollution and Carbon Reduction" and defining its accounting model [9] to provide suggestions on the top-level design of the system of "Collaborative Governance of Pollution and Carbon Reduction" [10–12]; From the path and measures to promote the synergy between Pollution and Carbon Reduction in an all-round energy transition [13] to the key path and policy research to provide ideas for the synergy between Pollution and Carbon Reduction [14]. Then, from the spatial auto-correlation and MGWR model in research methods [15] to the three urban agglomerations of Beijing-Tianjin-Hebei, Yangtze River Delta, and Pearl River Delta in the research object [16,17] studied the spatial evolution characteristics and regional heterogeneity of Pollution and Carbon Reduction from different regional levels. On account of the above studies, with accurately defining the concept of the coordination degree of Pollution and Carbon Reduction, this paper attempts to use the accounting model to measure the coordination degree of Pollution and Carbon Reduction in the Yangtze River Delta, Pearl River Delta, Beijing-Tianjin-Hebei, the Middle reaches of the Yangtze River and Chengdu-Chongqing urban agglomerations from 2016 to 2021, based on the consensus reached on the synergy between Pollution and Carbon Reduction. To explore the synergy between Pollution and Carbon Reduction in five urban agglomerations and their spatial heterogeneity, and further explore the influencing factors of the spatial differentiation of the Coordinated Degree of Pollution and Carbon Reduction in five urban agglomerations, this paper aims to analyze the spatial differences of five urban agglomerations and their causes, and eventually propose well-targeted adjustment policies for further improving and optimizing the synergy between Pollution and Carbon Reduction.

## Research design

### Selection of five national urban agglomeration

According to the government document [5], this paper selects five national urban agglomerations as research objects—Yangtze River Delta, Pearl River Delta, Beijing-Tianjin-Hebei, the Middle reaches of the Yangtze River and Chengdu-Chongqing urban agglomerations. The specific cities included in the national urban agglomerations are shown in Table 1. According to the development stage of urban agglomerations, the Yangtze River Delta and Pearl River Delta urban agglomerations can be classified as mature urban agglomerations, the Beijing-Tianjin-Hebei, the Middle reaches of the Yangtze River and Chengdu-Chongqing urban agglomerations can be classified as high-speed growth urban agglomerations, and the remaining unselected urban agglomerations are early-stage/mid-stage urban agglomerations [4]. As the rest urban agglomerations are backward in infrastructure development and do not form a regional synergistic governance system, the study significance of the synergistic effect of such urban agglomerations on pollution and carbon reduction is trivial, and thus five national urban agglomerations are taken as the target of the study.

**Table 1:** Five national urban agglomerations.

Urban agglomeration	Specific cities
the Yangtze River Delta urban agglomeration	Shanghai, Nanjing, Wuxi, Changzhou, Suzhou, Nantong, Yancheng, Yangzhou, Zhenjiang and Taizhou in Jiangsu Province, Hangzhou, Ningbo, Jiaxing, Huzhou, Shaoxing, Jinhua, Zhoushan and Taizhou in Zhejiang province, Hefei, Wuhu, Ma ’anshan, Tongling, Anqing, Chuzhou, Chizhou and Xuancheng in Anhui Province, etc
Pearl River delta urban agglomeration	"Guangfozhao" (Guangzhou, Foshan, Zhaoqing), "Shenzhen-Dongguan-hui" (Shenzhen, Dongguan, Huizhou), "Zhuzhongjiang" (Zhuhai, Zhongshan, Jiangmen) and other three new metropolitan areas
The Beijing-Tianjin-Hebei urban agglomeration	Beijing and Tianjin are two municipalities directly under the central government. They also include Baoding, Tangshan, Langfang, Shijiazhuang, Qinhuangdao, Zhangjiakou, Chengde, Cangzhou, Hengshui, Xingtai, Handan and Anyang in Henan Province
Middle reaches of Yangtze River urban agglomeration	Wuhan, Huangshi, Ezhou, Huanggang, Xiaogan, Xianning, Xiantao, Qianjiang, Tianmen, Xiangyang, Yichang, Jingzhou and Jingmen in Hubei Province, and Changsh, Zhuzhou, Xiangtan, Yueyang, Yiyang, Changde, Hengyang and Loudi in Hunan Province, Nanchang, Jiujiang, Jingdezhen, Yingtan, Xinyu, Yichun, Pingxiang, Shangrao, Fuzhou, Ji ’an in Jiangxi Province
Chengdu-Chongqing urban agglomeration	Chongqing Municipality and Chengdu, Zigong, Luzhou, Deyang, Mianyang, Suining, Neijiang, Leshan, Nanchong, Meishan, Yibin, Guang ’an, Dazhou, Ya ’an, Ziyang and other 16 cities in Sichuan Province

### Data sources for measuring the coordinated degree

The total amount of environmental pollutants (*SC*) is replaced by the total volume of municipal solid waste (MSW) in the five national urban agglomerations from 2016 to 2021, and the total amount of pollutant carbon emissions is replaced by the carbon dioxide equivalent generated by different disposal methods of MSW. The ratio of total municipal waste carbon emission to total municipal waste is utilized to calculate the Coordinated Degree of Pollution and Carbon Reduction in different regions [9].

#### (1) Data source of pollutant carbon emission equivalent

Municipal solid waste mainly includes four ways: incineration, landfill, composting, and anaerobic fermentation, corresponding to the total carbon emissions of different amount ranges. According to the life cycle theory, any food waste, household waste, or pollutant will produce a corresponding amount of carbon emission equivalent, in kgCO2eg/kg. Based on the data from the National Statistical Yearbook, the unit order of magnitude of household waste and carbon emissions increase 1,000 times year-on-year, namely tons of carbon dioxide equivalent per ton, or "teq/t" for short. The carbon emission equivalent of the four disposal methods of municipal sludge is used to replace the carbon emission of pollutants, and the carbon emission equivalent of pollutants with the same disposal methods is calculated as shown in Table 2.

**Table 2:** Carbon emission equivalent of pollutant and disposal method (teg/t).

Total pollutant carbon emission	Fuel *C*1	Electric power *C*2	Direct emission *C*3	Recycling *C*4	Total *SC*
*eq*1	2.4	0	420	0	422.4
*eq*2	271	31.5	61.4	-235	128.90
*eq*3	0	7.42	50.67	0	58.09
*eq*4	0	19.6	140	-310	-150.40

Note: Carbon emission equivalent in Table 2 is replaced by municipal sludge carbon emission.

#### (2) Data resource of disposal ratios

According to the report of city appearance and environmental sanitation in the *Statistical Yearbook of Urban and Rural Construction* (2016–2021), preprocess urban waste data, the domestic garbage removal volume, and the total amount of harmless disposal in the counties and cities included in the five major national urban agglomerations can be calculated. The *statistical yearbook* provides data on landfills, incineration, and others (including composting and anaerobic fermentation). For the sake of effectively calculating the comprehensive utilization of waste resource reuse and disposal, this paper sets the anaerobic fermentation ratio of 0.1% for the areas where the "other" column is not 0 in the statistical table. IF THEN ELSE (*c*, *d*, *e*), the conditional function of system dynamics, is applied to calculate the landfill ratio and anaerobic fermentation ratio. *c* is the condition and *d*, and *e* are the variable values of whether the condition is true:

Anaerobicfermentationratiok3=IFTHENELSE(Q3>0,0.001,0) (1)

Among them, *Q*3 is the total amount of harmless "other" treatments in each city cluster, and 0.001 is the anaerobic fermentation disposal ratio.


$$\mathrm{Compost}\;\mathrm{ratio}\;k3=IF\;THEN\;ELSE\;\left(Q3>0,\frac{Q3}{Dq}-0.001,0\right)$$ (2)

Incinerationratiok2=Q2/Dq (3)

Landfillratiok1=Q1/Dq (4)

#### (3) Proportion of disposal methods to pollution and carbon reduction

**Fig 1:**
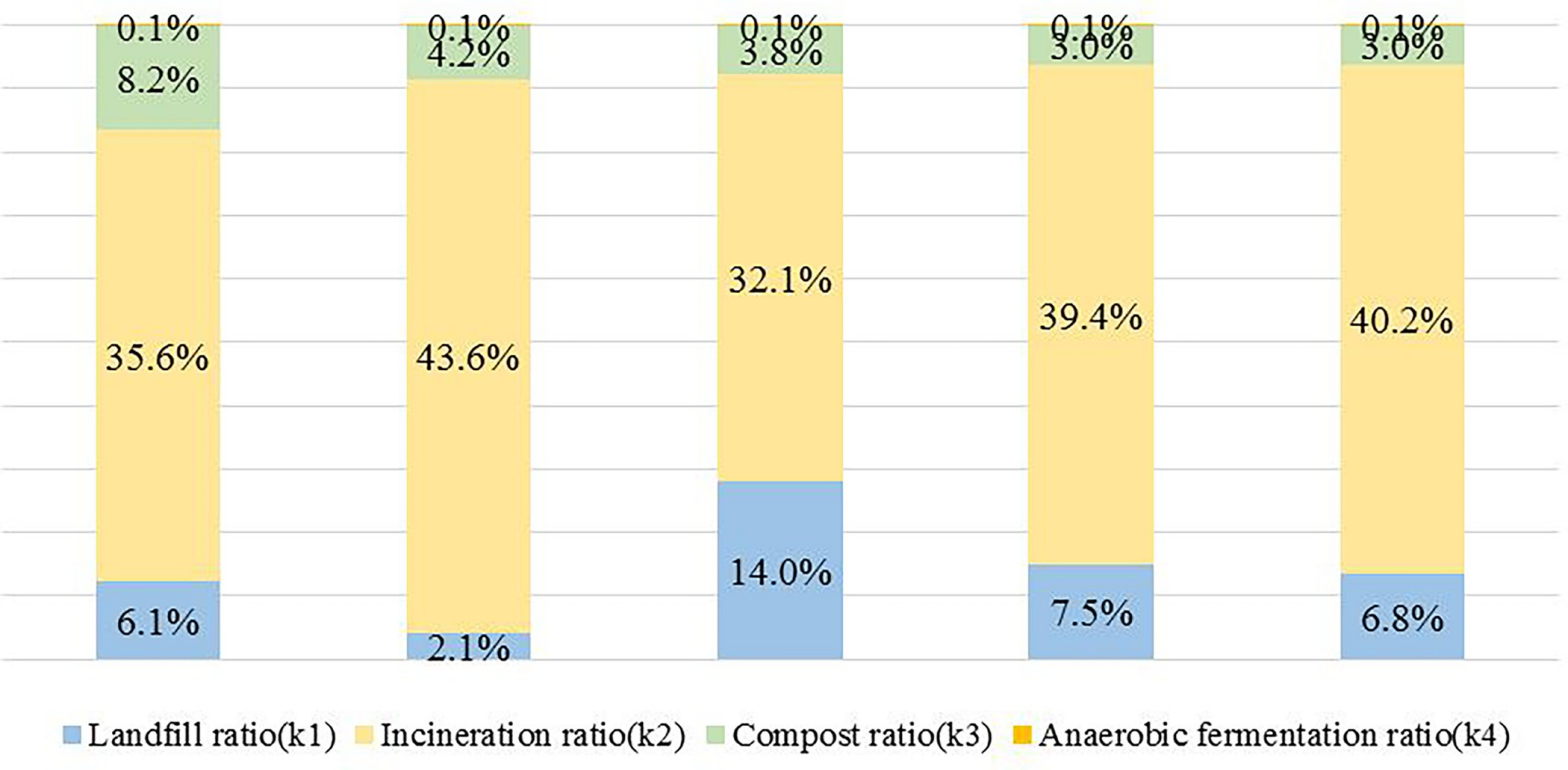
Proportion structure of carbon reduction methods.

According to Eqs (1)–(4), the proportion structure of carbon reduction disposal methods in five national urban agglomerations is drawn, as shown in Fig 1 (the urban agglomerations corresponding to each bar in Fig 1 are Beijing-Tianjin-Hebei, Yangtze River Delta, Middle Yangtze River, Pearl River Delta, and Chengdu-Chongqing urban agglomeration, respectively).

As shown in Fig 1, taking the data of the harmless disposal of municipal waste in the five national urban agglomerations in 2021 as an example, it can be found that among the five national urban agglomerations, the proportion of garbage incineration accounts for the major body, ranging between 30% and 45%. The proportion of landfill takes second place, which is 2.1% to 14.0% or so, with the landfill ratio of the Middle reaches of the Yangtze River being the largest. This reflects the relatively inferior construction of separate waste collection and separate transportation systems in this urban agglomeration, the weak capacity for waste incineration treatment, and the failure to form a sensible plan for waste disposal. These may contribute to hidden dangers such as environmental pollution in air, groundwater, and soil, as well as health threats to residents in this urban agglomeration. Two new ways of composting and anaerobic fermentation are put into trial use, so their share is still minor, but cannot be neglected.

### Method introduction and sample selection

To measure the coordination degree of Pollution and Carbon Reduction in the five urban agglomerations, the Coordination Degree of Pollution and Carbon Reduction should be defined first. This paper also selects the definition of the Coordinated Degree of Pollution and Carbon Reduction following the Implementation Plan of Carbon Reduction Synergy [9], namely: The ratio of the total carbon dioxide equivalent emitted by fuel consumption, power consumption, direct emission, and recycling of environmental pollutants that can produce carbon emissions in the process of harmless treatment, reduced treatment, low carbonation, and resource disposal to the total amount of environmental pollutants [8]. The smaller the ratio, the better the Coordinated Degree of Pollution and Carbon Reduction. Consequently, the accounting model of Coordinated Degree of Pollution and Carbon Reduction is established as follows (Eq 5):

$$Sd=\frac{Total\;carbon\;emissions\;of\;pollutants\;(SC)}{100\times total\;amount\;of\;pollutant\;(Dq)}$$ (5)

Where: Total pollutant (*Dq*) = landfill (*Q*1) + incineration (*Q*2) + compost (*Q*3) + recycling (*Q*4);

Total pollutant carbon emission (*SC*) = landfill (*Q*1) × landfill equivalent (*eq*1) + incineration (*Q*2) × incineration equivalent (*eq*2) + compost (*Q*3) × compost equivalent (*eq*3) + recycling amount (*Q*4) × resource equivalent (*eq*4).

The Coordinated Degree of Pollution and Carbon Reduction is a numerical ratio, so it is dimensionless. The measurement unit of total carbon emission *SC* is one million tons (Mt), and the quantization unit of total pollutant *Dq* is ten thousand tons (10kt). The denominator is given a multiplier of 100 to optimize the ratio, to compare the change of Coordinated Degree of Pollution and Carbon Reduction and regional differentiation more clearly and intuitively.

### Measurement of the coordinated degree

**Fig 2:**
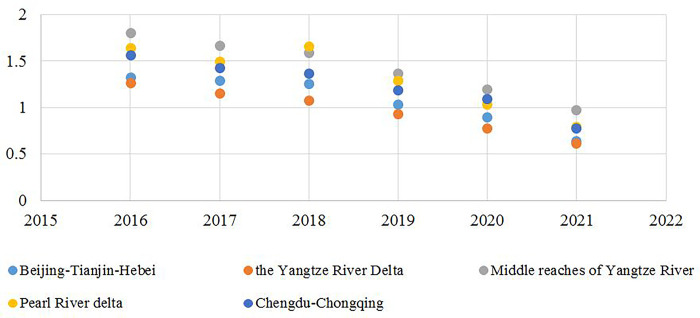
Coordinated degree from 2016 to 2021.

The function INTEG (a, b), representing the accumulation change, is used to calculate the Coordinated Degree of Pollution and Carbon Reduction, where *a* is the change amount of the variable and *b* is variable. Per Eq (5), the Coordinated Degree of Pollution and Carbon Reduction of the five national urban agglomerations is calculated, as shown in Fig 2.

It can be seen from Fig 2 that the five national urban agglomerations all have a highly Coordinated Degree of Pollution and Carbon Reduction, among which the Middle reaches of the Yangtze River, the Pearl River Delta, and Chengdu-Chongqing urban agglomerations have the best Coordinated Degree of Pollution and Carbon Reduction. Compared with the first three, the Coordinated Degree of Pollution and Carbon Reduction in the Beijing-Tianjin-Hebei and Yangtze River Delta urban agglomerations is slightly lower. There is a small difference between the total pollutant carbon emission of the Yangtze River Delta and the other four urban agglomerations, while the total pollutant emission reaches 102,094,100 tons, indicating that the rapid economic development of the Yangtze River Delta leads to greater pressure on environmental pollution control. From the longitudinal analysis of the time trend, the Coordination Degree of Pollution and Carbon Reduction in the five urban agglomerations shows a fluctuating trend, first decreasing and then increasing from 2016 to 2018, and presents a steady decline from 2019 to 2021. From the horizontal comparison of the five urban agglomerations, compared with other urban agglomerations, which maintain stability and then decline, the Pearl River Delta urban agglomerations had a peak of 1.662 in 2018, reflecting the high coordination of this economic development region.

**Fig 3:**
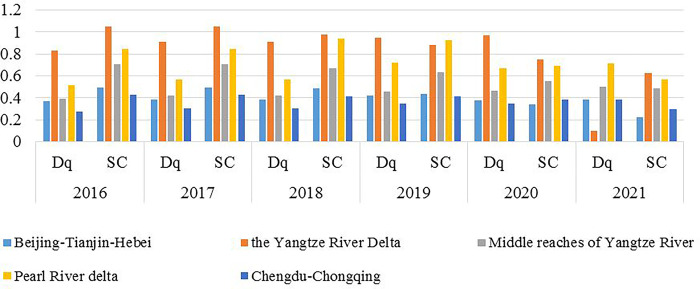
*SC* and *Dq* of urban agglomerations from 2016 to 2021.

Based on the calculation of the Coordination Degree of Pollution and Carbon Reduction in five national urban agglomerations, the variation trend of total pollutant amount *Dq* and total pollutant carbon emission *SC* in each urban agglomeration is further analyzed (Fig 3). After 2018, when the Coordinated Degree of Pollution and Carbon Reduction in Beijing-Tianjin-Hebei, Yangtze River Delta, Pearl River Delta, the Middle reaches of the Yangtze River and Sichuan-Chongqing urban agglomerations declined, the total carbon emission of pollutants also decline steadily, but the total amount of pollutants shows a trend of fluctuating growth. As the Coordinated Degree is defined as the ratio of total municipal waste carbon emission to total municipal waste, it is speculated that the reduction of Coordinated Degree of Pollution and Carbon Reduction in the five urban agglomerations is caused by the combination of a stable decline in carbon emissions and fluctuating increase in pollutants. Nevertheless, for the aim of sustainable development, the amount of pollution emissions ought to be controlled. Consequently, to depress the coordinated degree, the technology of processing pollutants should be improved to reduce carbon emissions per unit of pollutant. Meanwhile, the total carbon emission has not increased year-on-year, indicating that all regions attach great importance to environmental governance on the whole, and the discrepancy between regions has increased.

## Spatial differentiation of coordinated effects

### Measurement method of spatial differentiation

Theil Index (TB) and coefficient of variation (CV) [16] are used to calculate and explore the heterogeneity of Coordinated Degree of Pollution and Carbon Reduction among different national urban agglomerations. The calculation formula is shown in Eq (6) and Eq (7):

$$TB=\frac{1}{s}\sum_{i=1}\frac{y_{m}}{u}ln\frac{{\bar{y}}_{m}}{u}$$ (6)

$$CV=\frac{\sqrt{\frac{1}{s}\sum_{m=1}^{s}{\left(x_{m}-{\bar{x}}_{m}\right)}^{2}}}{{\bar{x}}_{m}}$$ (7)

Where *s* represents the number of samples; *x*_*m*_, *y*_*m*_ and ${\bar{x}}_{m}$, ${\bar{y}}_{m}$ represent a sample index and the average of sample parameter values of sample *m* respectively; In this paper, *TB* is further decomposed into intra-city agglomeration gap and inter-city agglomeration gap, and the decomposition method is shown in Eq (8) [17]:

$$TB=T_{BL}+T_{BD}=\sum_{q}^{5}f_{q}ln\frac{u_{q}}{u}+\sum_{q}^{5}f_{q}\frac{u_{q}}{u}ln\frac{u_{q}}{u}$$ (8)

Where *u*_*q*_ represents the Coordinated Degree of Pollution and Carbon Reduction of the *q*th urban agglomeration; *u* represents the average of Coordinated Degree of Pollution and Carbon Reduction; *f*_*q*_ is the ratio between the number of cities in the *q*th urban agglomeration and the total number of cities; *T*_BL_ represents differences within groups; *T*_BD_ indicates differences between groups.

### Measuring method of regional linkage strength of synergy

The spatial linkage gravity model [16,18,19] is utilized to calculate and explore the regional linkage strength of the Coordinated Degree of Pollution and Carbon Reduction among the five national urban agglomerations, to measure the interaction between cities within the urban agglomerations on the Coordinated Degree of Pollution and Carbon Reduction. It reflects the radiation capacity of any urban agglomeration to the surrounding region and the acceptance degree of the surrounding region to the radiation capacity of the urban agglomeration [20]. The calculation formula is shown in Eq (9):

$$R_{ab}=K\frac{{Sd}_{a}{Sd}_{b}}{T_{ab}^{2}}$$ (9)

Where *R*_*ab*_ represents the regional linkage strength of the Coordinated Degree of Pollution and Carbon Reduction between urban agglomerations *a* and *b*; *Sd*_*a*_ and *Sd*_*b*_ represent the Coordinated Degree of Pollution and Carbon Reduction in urban agglomerations *a* and *b*; *T*_*ab*_ represents the distance between urban agglomeration (unit: km); *K* is the gravitational constant, usually 1.

### Spatial differentiation of synergy between pollution and carbon reduction

#### Theil index difference of coordinated degree

The results obtained by Eqs (6) to (9) are shown in Table 3. It can be deduced that:

**Table 3:** Theil index and its decomposition of coordinated degree.

Year	1	2	3	4	5	Intra-regional differences	Difference between regions	Contribution rate	
								Intra-regional	Between regions
2016	0.281	0.573	0.581	0.176	0.321	0.322	0.064	83.39%	16.61%
2017	0.275	0.575	0.581	0.178	0.323	0.322	0.064	83.40%	16.60%
2018	0.276	0.590	0.592	0.168	0.328	0.327	0.064	83.74%	16.26%
2019	0.278	0.581	0.583	0.174	0.322	0.324	0.064	83.50%	16.50%
2020	0.278	0.590	0.577	0.180	0.312	0.323	0.064	83.48%	16.52%
2021	0.281	0.573	0.581	0.176	0.321	0.322	0.064	83.39%	16.61%

Note: 1, 2, 3, 4, and 5 represent the five urban agglomerations of Beijing-Tianjin-Hebei, the Yangtze River Delta, the Middle reaches of Yangtze River, Pearl River Delta, and Chengdu-Chongqing, respectively.

The Thiel index of the Coordinated Degree of the two urban agglomerations in the Yangtze River Delta and the Middle reaches of the Yangtze River is above 0.5, indicating that the regional differences in carbon reduction synergy are large. This may be due to the reason that the urban agglomerations in the Yangtze River Delta and the Middle reaches of the Yangtze River contain more provinces and cities, including 26 and 31 cities respectively, and the inter-provincial connection is not close enough, making integration and coordination tough. Therefore, the discrepancy between the Coordinated Degree of Pollution and Carbon Reduction is significantly greater than that of the other three urban agglomerations. The Yangtze River Delta and the Middle reaches of the Yangtze River have relatively fast economic development. In particular, the Yangtze River Delta urban agglomeration plays a demonstration role in regional economic innovation and multi-industrial integration and strengthens its ties through inter-regional economic cooperation, trade exchanges, and preferential policies. Consequently, the difference in Coordinated Degree between urban agglomerations and other urban agglomerations is smaller than that between different prefecture-level cities within urban agglomerations.

The Theil Indexes of Coordinated Degree of Pollution and Carbon Reduction in Beijing-Tianjin-Hebei, Pearl River Delta, and Chengdu-Chongqing urban groups are all less than 0.33, indicating that there is little difference in the collaborative governance of Pollution and Carbon Reduction within the region, which is speculated to be due to the relatively small number of cities covered by them, the small geographical distance within the urban groups, the similar regional culture, the similar development path, the sharing of regional resources, and the frequent inter-regional connections, so the differences in the collaborative governance of Pollution and Carbon Reduction are small. Among them, the Theil Index of Coordinated Degree of Pollution and Carbon Reduction in the Pearl River Delta is the smallest, which is between 0.168–0.180. This may be because the Pearl River Delta urban agglomeration only includes nine cities: Guangzhou, Foshan, Zhaoqing, Shenzhen, Dongguan, Huizhou, Zhuhai, Zhongshan, and Jiangmen, and it has the smallest regional coverage area, with well-developed transportation networks such as roads and railways and convenient coordination. That’s why it is the most closely connected. As an urban agglomeration highly open to the outside world and under the jurisdiction of one province, the Pearl River Delta is superior to the Yangtze River Delta and Beijing-Tianjin-Hebei region in terms of resource integration and coordination. The latter region is under the jurisdiction of multiple provinces and cities, and the operation of integration and coordination is more complicated. This factor enables the Pearl River Delta to better conduct collaborative governance of Pollution and Carbon Reduction under unified planning and arrangement, give play to the merits of diverse cities, cooperate, and promote the virtuous cycle of urban agglomeration.

From the longitudinal analysis, the Theil Index of the Coordinated Degree of Pollution and Carbon Reduction in the Yangtze River Delta, the Middle reaches of the Yangtze River, the Pearl River Delta, and the Sichuan-Chongqing urban agglomerations show a trend of first rising and then declining in the past 6 years, indicating that the differences of the Coordinated Degree of Pollution and Carbon Reduction in these urban agglomerations show a trend of decreasing gradually, while the variation trend of the Theil Index of the Coordinated Degree of Pollution and Carbon Reduction in the Beijing-Tianjin-Hebei urban agglomerations is opposite, and the discrepancies of regional governance increase. On the whole, the contribution rate of intra-regional differences is about 83%, much higher than the contribution rate of inter-regional differences. Consequently, the difference in the Coordinated Degree of urban agglomerations in Pollution and Carbon Reduction is mainly caused by intra-regional differences.

#### Coefficient of variation of coordinated degree

**Fig 4:**
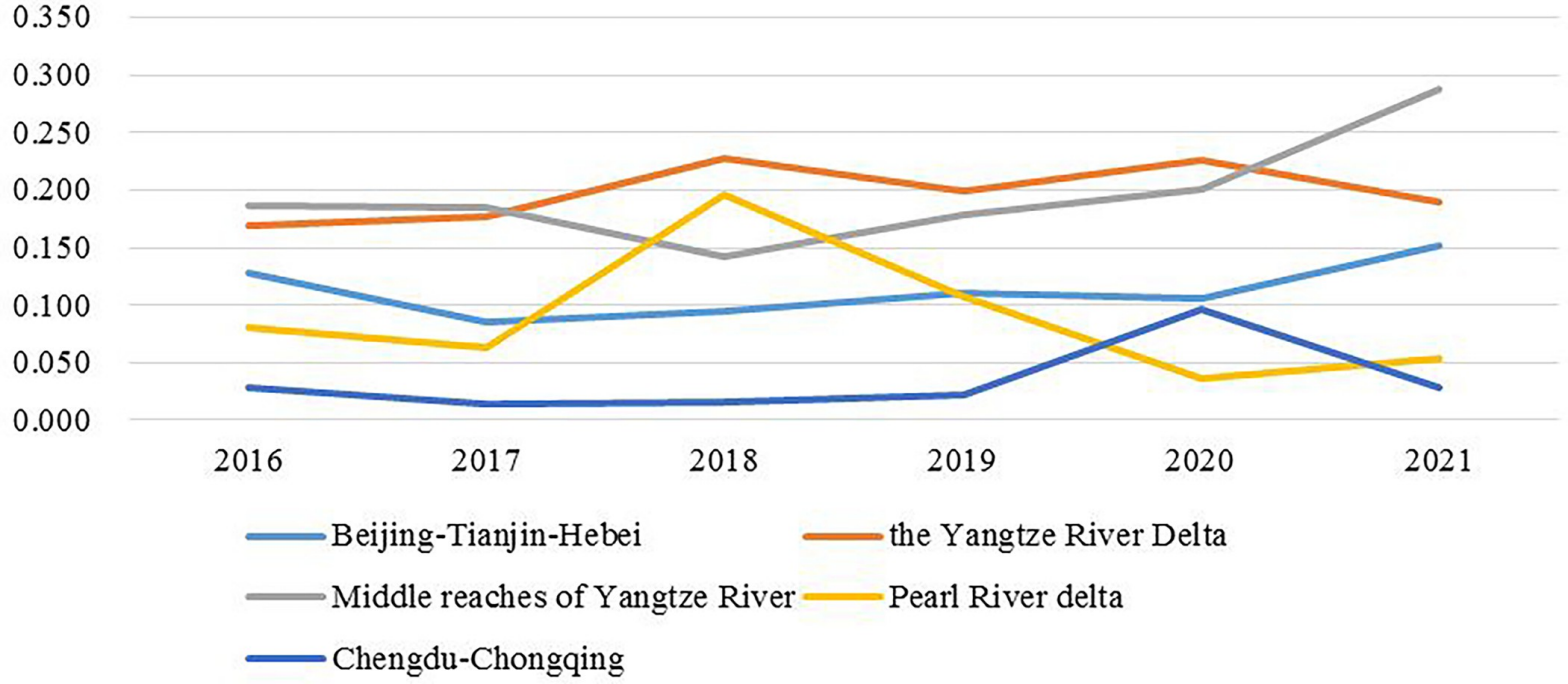
Coefficient of variation of coordinated degree.

Eq (7) is used to calculate the variation coefficient of the Coordinated Degree of Pollution and Carbon Reduction for the five national urban agglomerations. The calculated results are shown in Fig 4. It can be intuitively found that the coefficient of variation of the five national urban agglomerations has a large discrepancy in the fluctuation range. The coefficient of variation of urban agglomerations in the Middle reaches of the Yangtze River and the Yangtze River Delta is relatively high on the whole, and the coefficient of variation of urban agglomerations in the Middle reaches of the Yangtze River reaches the peak value of 0.287 in 2021, which may be because the urban agglomerations in the Middle reaches of the Yangtze River span the largest number of provinces and cities (3 provinces and 31 cities), the vast coverage area, and the incomplete transport network system in the region, so the resource coordination is hindered to a certain extent. Moreover, the economic development level of the cities in the Middle reaches of the Yangtze River urban agglomeration differs greatly, and the degree of unification and coordination of its development is smaller than that of other urban groups. There is a lack of a unified policy system for promoting the collaborative governance of Pollution and Carbon Reduction among different provinces and cities, so the regional differences in the synergy between Pollution and Carbon Reduction are large. The overall coefficient of variation of the Beijing-Tianjin-Hebei urban agglomeration is the second highest, experiencing a decline in 2017 and then increasing slowly. This may be because the urban agglomeration spans three provinces, and the coordinated control of inter-regional environmental pollution involves the cooperation of several local governments. Additionally, the Beijing-Tianjin-Hebei urban agglomeration, as the "world-class urban agglomeration" planning area formulated by The State Council, actively promotes regional connections within it, but disparate provinces and cities retain their distinct development characteristics to a certain extent, so there are certain differences in collaborative governance. The coefficient of variation in the Pearl River Delta and Sichuan-Chongqing urban agglomerations is relatively low, and their values are 0.053 and 0.028 respectively in 2021. It can be predicted that the number of inner cities in the Pearl River Delta and Sichuan-Chongqing urban agglomerations is only 9 and 16, indicating that these two urban agglomerations contain few cities and are more convenient for integration and coordination. The coefficient of variation in the Pearl River Delta urban agglomeration reached its peak in 2018 and then declined rapidly. Although it rebounded in 2021, its overall level has dropped to a low level. To further reasonableness, the transportation network of the Pearl River Delta urban agglomeration is developed, owing to the geographical advantages of Guangdong, Hong Kong, and Macao, natural and convenient cultural and economic links, production factors like capital, talent, and technology are gathered, so the differences in the collaborative governance of Pollution and Carbon Reduction are small. The coefficient of variation of Sichuan-Chongqing urban agglomeration is the lowest, relying on the radiating driving effect of the Chengdu metropolitan area and Chongqing metropolitan area, this urban agglomeration forms a development pattern of "one axis, five belts, two cores, and three districts", with strong intensity of urban planning and construction management and strong binding effect of urban planning, so it has a high degree of synergy and small regional differences in environmental governance. Generally, the coefficient of variation of the five national urban agglomerations is less than 0.3, and the coordinated degree of Pollution and Carbon Reduction is considerable. As the national key economic development planning area, it has an outstanding demonstration effect in environmental governance coordination.

### Regional relationship of synergy

The spatial linkage gravity model is applied to calculate the regional linkage degree of the collaborative governance of Pollution and Carbon Reduction within the five national urban agglomerations, and the results are shown in Table 4.

**Table 4:** Spatial linkage intensity of pollution and carbon reduction.

Urban agglomeration	Distance between urban agglomerations(km)	Spatial linkage strength of collaborative governance of Pollution and Carbon Reduction	
1--2	998.88	R12	3.927E-07
1--3	1145.57	R13	6.231E-07
1--4	1754.85	R14	5.101E-07
1--5	1239.3	R15	4.976E-07
2--3	801.57	R23	5.952E-07
2--4	1182.92	R24	4.873E-07
2--5	1249.69	R25	4.754E-07
3--4	613.57	R34	7.732E-07
3--5	496.44	R35	7.542E-07
4--5	849.46	R45	6.17502E-07

Note: In Table 4, numbers 1, 2, 3, 4, and 5 are substituted for the Beijing-Tianjin-Hebei, Yangtze River Delta, Middle reaches of the Yangtze River, Pearl River Delta, and Chengdu-Chongqing urban agglomerations.

From the analysis of Table 4, it can be deduced that the Beijing-Tianjin-Hebei and Yangtze River Delta urban agglomerations have a strong radiating driving effect on the surrounding areas, mainly caused by the core radiating effect of Beijing and Tianjin in the Beijing-Tianjin-Hebei region, and Shanghai, Hangzhou, and Ningbo in the Yangtze River Delta. Due to the influence of the integration policy of the Yangtze River Delta, the environmental pollution control level of the Yangtze River Delta urban agglomeration has been improved, and collaborative governance among cities has been promoted. As an old saying goes, "granaries are full and courtesy is known", while the urban agglomeration achieves high-quality economic development and promotes the realization of a green and low-carbon economy, the local government has signed Key Projects for Matching Cooperation with the government of Hunan, Jilin, and Hainan provinces to help improve the city appearance and strive for a national model. Led by Beijing, the Beijing-Tianjin-Hebei city cluster will optimize its spatial pattern through the orderly distribution of non-capital functions to further promote the coordinated development of Beijing-Tianjin-Hebei and bring benefits to other provinces and cities. Relatively speaking, the link between the Middle reaches of the Yangtze River, the Pearl River Delta, the Sichuan-Chongqing urban agglomeration, and the surrounding areas is weak, and may be caused by geographical location, coverage area, local culture, and other factors.

## Research conclusions and implications

### Research conclusions

In this paper, based on the definition of the synergy of Pollution and Carbon Reduction, as well as the accounting model of the Coordinated Degree of Pollution and Carbon Reduction constructed and the statistical data from 2016 to 2021, the Coordinated Degree of Pollution and Carbon Reduction of five national urban agglomerations is calculated, and its dynamic trend and spatial distribution are analyzed. Furthermore, the spatial differentiation of Pollution and Carbon Reduction degree in five urban agglomerations is analyzed, and the regional linkage strength of the Coordinated Degree of Pollution and Carbon Reduction among five urban agglomerations is deduced. The findings include:

First, on the whole, compared with the Coordinated Degree of the five urban agglomerations from 2016 to 2020, the average annual fluctuation range of the Coordinated Degree of the five national urban agglomerations in 2021 is smaller, falling between 17.71% to 21.31%. Additionally, the urban agglomeration of the Middle reaches of the Yangtze River has the highest degree of Pollution and Carbon Reduction in 2021, followed by the Pearl River Delta and the Yangtze River Delta.

Second, from the perspective of time series analysis, the Coordinated Degree of Pollution and Carbon Reduction in the five urban agglomerations show a downward trend on the whole. From 2016 to 2018, it showed a fluctuation trend of first decreasing and then increasing, and from 2019 to 2021, it presented a steady decline. From the horizontal comparison of the five urban agglomerations, compared with other urban agglomerations, which maintained a stable trend and then declined, the Pearl River Delta urban agglomerations had a peak of 1.662 in 2018, reflecting the high coordination of this economic development region.

Third, by further analyzing the internal composition of the coordination degree of Pollution and Carbon Reduction of the five urban agglomerations, namely the variation trend of the total amount of pollutants and carbon emissions, it can be concluded that when the coordination degree of Pollution and Carbon Reduction of the five major national urban agglomerations decreases overall, which is mainly caused by the comprehensive effect of steady decreases of the total amount of carbon emissions and the increases of the total amount of pollutants. Besides, although the total amount of pollutant emissions increase due to industry expansion and other reasons these years, the government has put forward documents to reduce pollutants to guarantee environmental sustainability. Therefore, the technology of garbage disposal should be improved to whittle down the coordinated degree.

Fourth, the analysis of the intra-regional differences in the coordination degree of Pollution and Carbon Reduction in the five national urban agglomerations shows that there are large intra-regional differences in the Yangtze River Delta and the Middle reaches of the Yangtze River. The Theil index is above 0.5, which may be because the Yangtze River Delta and the Middle reaches of the Yangtze River contain more provinces and cities, including 26 and 31 cities respectively, and the inter-provincial connection is not close enough. Therefore, it is tough to integrate and coordinate. The Theil indexes of the other three urban agglomerations are less than 0.33, with subtle differences within the region. Among them, the Theil index of the Pearl River Delta urban agglomeration is the smallest, ranging from 0.168 to 0.180 in the five years from 2016 to 2021, which may be due to the small number of prefecture-level cities and the smallest regional coverage area, as well as the developed and convenient coordination of road, railway, and other transportation networks.

Fifth, the Middle reaches of the Yangtze River and the Yangtze River Delta urban agglomerations have a higher level of coefficient of variation, which may be caused by the wide regional coverage area, large differences in transportation network systems, and large differences in resource allocation. The coefficient of variation of Coordinated Degree in Beijing-Tianjin-Hebei urban agglomeration is the second, and the Pearl River Delta and Sichuan-Chongqing urban agglomeration have low levels of variation, which may be related to the fact that the number of cities in the Pearl River Delta and Sichuan-Chongqing urban agglomeration is only 9 and 16, convenient for integration and coordination.

### Suggestions for countermeasures

The results of the above study show that: the Coordinated Degree of the five national urban agglomerations is low, there is a great discrepancy in the Coordinated Degree within urban agglomerations, and a deeper mechanism of action has yet to emerge. The collaborative governance of Pollution and Carbon Reduction should be made in the direction of policy, science, technology, etc., and promote the improvement of environmental quality and high-level economic quality.

#### First, guide policy innovation through coordination

Pollution and Carbon Reduction have the same target, and target synergy is the key to deciding the policy system of collaborative governance. Given the low coordination degree caused by the independent and different modes of collaborative governance systems for Pollution and Carbon Reduction in urban agglomerations, it is essential to effectively implement the national " Carbon peak, Carbon neutrality" goals, improve the system and mechanism for environmental pollution control and addressing climate change, and innovate the coordinated policy system for Pollution and Carbon Reduction targets in urban agglomerations. The Beijing-Tianjin-Hebei city cluster should give full play to the guiding role of government policies, strengthen the reduction of carbon emissions and realize the green transformation of economic and social development. The Yangtze River Delta urban agglomerations should improve climate change investment and financing policies, and strengthen the spatial linkages within urban agglomerations. The Pearl River Delta urban agglomeration should further improve its environmental management mechanism, and innovate coordinated incentive policies for Pollution and Carbon Reduction.

#### Second, achieve synergy through technological innovation

Encourage foreign investment to invest in low-carbon green high-tech enterprises, introduce, learn, and absorb foreign advanced manufacturing technologies. In terms of environmental pollution control, set up a special research and development fund for the collaborative treatment of Pollution and Carbon Reduction technologies, carry out research and development of key technologies, and improve the scientific and technological innovation mechanism for the collaborative treatment of Pollution and Carbon Reduction in urban agglomerations, to provide a strong scientific and technological guarantee for realizing the synergy between Pollution and Carbon Reduction. In terms of resource utilization, promote the application of existing successful experiences and technologies in reducing pollution and carbon, strengthen the research and development and application of collaborative regulation technologies, optimize the selection of technological routes for Pollution and Carbon Reduction, and improve resource utilization efficiency.

#### Third, promote regional connectivity through win-win cooperation

Since greenhouse gases and pollutant emissions are of the same root, same origin, and all industries and fields within urban agglomerations are responsible for environmental pollution and greenhouse gases, all urban agglomerations should, based on their regional advantages and the concept of coordinated development, adopt coordinated governance strategies for Pollution and Carbon Reduction based on local conditions, and jointly promote the high-quality integrated development of the regional economy.

Specifically, the Beijing-Tianjin-Hebei urban agglomeration should promote green manufacturing in the industrial field, accelerate the green transformation of steel, petrochemical, building materials, and other industries, and strictly control the construction of projects with high energy consumption and high emissions. The Yangtze River Delta urban agglomeration should optimize the allocation of regional human capital, strengthen cooperation among cities, and promote the continuous improvement of ecological environment quality. The Pearl River Delta urban agglomeration should continuously strengthen cooperation in industrial restructuring, industrial transfer, energy conservation, clean energy replacement, and technological upgrading. The Chengdu-Chongqing urban agglomeration should advocate the green, high-end, and intelligent development of traditional industries such as steel, machinery, chemical industry, and building materials, and concentrate on ecological protection, green economy, and resource recycling as an innovation ecosystem. The Middle reaches of the Yangtze River urban agglomeration should inherit the advantages of industrial gradient transfer. Concerted efforts ought to be made to bring in scientific and technological innovation factors and to stimulate high-end and high-quality enterprises to go global.

## Supporting information

S1 Appendix Data processing. (XLS)
